# MiR-449b-5p Ameliorates Hypoxia-induced Cardiomyocyte Injury through Activating PI3K/AKT Pathway by Targeting BCL2L13

**DOI:** 10.1007/s12010-024-04931-5

**Published:** 2024-04-06

**Authors:** Yang Jiang, Zeyan Liu, Li Ye, Jinglin Cheng, Jun Wan

**Affiliations:** Department of Emergency Medicine, The Second Affiliated Hospital of Anhui Medical University, No. 678 Furong Road, Jingkai District, Hefei, Anhui 230000 China

**Keywords:** Hypoxia, AC16 cardiomyocytes, Mir-449b-5p, BCL2L13, PI3K/AKT

## Abstract

Recent reports show miR-449b-5p reduces liver and renal ischemia/reperfusion (I/R) injury, but its effects on hypoxia-induced cardiomyocyte injury in ischemic heart disease are still unknown. In this study, AC16 human cardiomyocytes underwent hypoxic conditions for durations of 24, 48, and 72 h. We observed that miR-449b-5p expression was significantly downregulated in hypoxic AC16 cardiomyocytes. Elevating the levels of miR-449b-5p in these cells resulted in enhanced cell survival, diminished release of LDH, and a reduction in cell apoptosis and oxidative stress using CCK-8, LDH assays, flow cytometry, TUNEL staining, and various commercial kits. Conversely, reducing miR-449b-5p levels resulted in the opposite effects. Through bioinformatics analysis and luciferase reporter assays, BCL2-like 13 (BCL2L13) was determined to be a direct target of miR-449b-5p. Inhibiting BCL2L13 greatly inhibited hypoxia-induced cell viability loss, LDH release, cell apoptosis, and excessive production of oxidative stress, whereas increasing BCL2L13 negated miR-449b-5p’s protective impact in hypoxic AC16 cardiomyocytes. Additionally, miR-449b-5p elevated the levels of the proteins p-PI3K, p-AKT, and Bcl-2, while decreasing Bax expression in hypoxic AC16 cardiomyocytes by targeting BCL2L13. In summary, the research indicates that the protective effects of miR-449b-5p are facilitated through the activation of the PI3K/AKT pathway, which promotes cell survival, and by targeting BCL2L13, which inhibits apoptosis, offering a potential therapeutic strategy for ischemic heart disease by mitigating hypoxia-induced cardiomyocyte injury.

## Introduction

Acute myocardial infarction (AMI), a prevalent form of ischemic heart disease globally, is primarily marked by cardiomyocyte dysfunctions due to acute ischemia-hypoxia [1, 2]. This dysfunction is attributed to key factors such as apoptosis, necroptosis, and mitochondrial damage [3, 4]. In hypoxic conditions, these damaged mitochondria often release an excess of reactive oxygen species and show reduced activity in antioxidant enzymes [5]. Therefore, comprehending the molecular mechanisms driving hypoxia-induced cardiomyocyte dysfunction is crucial for developing new treatments for AMI patients.

MicroRNAs (miRNAs/miRs), tiny noncoding single-stranded RNAs approximately 22 nucleotides in length, suppress gene expression after transcription by attaching to the 3′-untranslated regions (3′-UTRs) of their target genes [6]. There is substantial evidence that various miRNAs, abnormally expressed, are linked to cardiovascular diseases including myocardial infarction, hypertrophy, and ischemia-reperfusion [7, 8]. For instance, inhibition of miR-141-3p diminishes apoptosis induced by hypoxia in H9C2 cardiomyocytes through the activation of the RP105-mediated PI3K/AKT pathway [9]. Elevating miR-194-5p levels protects against hypoxia/reoxygenation injury in cell cultures and cardiac ischemia/reperfusion injury in live models by reducing cardiac apoptosis and oxidative stress. This effect is achieved by targeting MAPK1 via the PTEN/AKT pathway [10]. Tan et al. [11] found that miR-145-5p/(NADPH) oxidase homolog 1 helps protect against myocardial ischemia/reperfusion (I/R) injury by reducing inflammation and apoptosis. MiR-449b-5p, recognized for its tumor-suppressive role in various cancers, such as nasopharyngeal carcinoma [12] and lung adenocarcinoma [13]. Importantly, miR-449b-5p has demonstrated its potential in ameliorating hepatic I/R injury by targeting HMGB1 and deactivating the NF-κB pathway, as elucidated by Zhang et al. [14]. The research conducted by Hu et al. [15] has underscored the significance of miR-449b-5p in the treatment of hepatic injury and its potential as a valuable resource for effectively mitigating liver disorders. Moreover, in the context of renal I/R injury, low miR-449b-5p expression and its subsequent overexpression have been associated with reduced inflammation and apoptosis in HK-2 cells [16]. Adding to its versatility, a recent study by Li et al. [17] has connected altered miR-449b-5p expression to the regulation of hepatic lipid metabolism, particularly through its influence on mitochondrial-cytoplasmic distribution. Additionally, the use of anti-miR-449b or APN has demonstrated effectiveness in maintaining cardiac Nrf-1 expression, alleviating cardiac oxidative stress, decreasing apoptosis, reducing the size of infarcts, and improving cardiac performance [18]. Given its involvement in I/R injury, it is plausible that miR-449b-5p could also have a crucial regulatory function in the dysfunction of cardiomyocytes induced by hypoxia.

BCL2-like 13 (BCL2L13), an Atg32 functional equivalent, possesses a single transmembrane domain at its C-terminus, four BCL2 homology domains (BH1-4), and a pair of WXXL/I motifs situated at the positions 147–150 and 273–276 [19]. This protein is involved in numerous physiological functions, including growth, development, and energy metabolism, and its imbalance has been linked to several pathologies like cardiovascular and degenerative diseases [20]. For example, BCL2L13 functions as an inhibitory factor in the reduction of ischemic injury by miR-874-3p, positioning it as a viable therapeutic target for ischemic stroke [21]. It was found to be overexpressed in cerebral I/R injury in rats and in oxygen-glucose deprivation/reoxygenation (OGD/R) affected SK-N-SH and IMR-32 cells, contributing to cerebral I/R injury [22]. On the contrary, a study by Zhang et al. [23] showed that SNHG15, by acting as a sponge for miR-141-3p, enhances BCL2L13 expression, thereby inhibiting IL-1β-induced apoptosis in chondrocytes and preventing articular cartilage damage. Our bioinformatics analysis indicates that BCL2L13 is a probable downstream target of miR-449b-5p. Given these findings, we have formulated the hypothesis that miR-449b-5p plays a significant role in hypoxia-induced cardiomyocyte dysfunction by targeting BCL2L13.

To substantiate our hypothesis, we established a cellular model of hypoxia-induced cardiomyocyte dysfunction using AC16 cardiomyocytes. This model allowed us to investigate the functional contributions of miR-449b-5p and BCL2L13 in hypoxia-induced apoptosis and oxidative stress. Furthermore, we explored the interplay between miR-449b-5p and BCL2L13 in the context of cardiomyocyte injury triggered by hypoxia.

## Materials and Methods

### Reagents

AC16 human cardiomyocytes, sourced from the BeNa Culture Collection (Beijing, China). MiR-449b-5p mimics, its inhibitor, negative control (NC), si-BCL2L13, si-NC, and BCL2L13-overexpressing plasmid (pcDNA3.1-BCL2L13) and vector control were purchased from Ribo Biological Co., Ltd. (Guangzhou, China). CCK-8 reagent (Beyotime, Shanghai, China), LDH Kit (Beijing Solarbio Science & Technology Co., Ltd.) and FITC-Annexin V/PI kit (BD Biosciences, San Jose, CA, USA) were purchased from corresponding companies. MDA (ab287797), ROS (ab287839), SOD (ab178012), specific primary antibodies against BCL2L13 (ab203516), Bcl-2 (ab196495), Bax (ab182733), PI3K (ab133595), p-PI3K (ab182651), AKT (ab8805), p-AKT (ab8933) and GAPDH (ab9485) were provide by Abcam (Cambridge, UK). Horseradish peroxidase-conjugated secondary antibodies (SC-2054 SC-2054) was purchased from Santa Cruz Inc., (Santa Cruz, CA, USA). RIPA protein lysate was from Thermo Fisher Scientific, Inc.) and BCA protein concentration kit was from Beyotime. Other inorganic reagents were purchased from Sigma-Aldrich.

### Cell Culture and Hypoxia Treatment

AC16 human cardiomyocytes were grown in DMEM medium (provided by Thermo Fisher Scientific), supplemented with 10% fetal bovine serum (from HyClone) and 1% penicillin/streptomycin (sourced from Sigma-Aldrich), and kept at a temperature of 37 °C. To induce hypoxia, AC16 cardiomyocytes were grown in a hypoxic incubator under conditions with 5% CO_2_, 1% O_2_, and 94% N_2_ for durations of 0 h, 12 h, 24 h, and 48 h. For the control group in normoxic conditions, cells were incubated in an environment with 5% CO_2_, 21% O_2_, and 74% N_2_. To create the in vitro model of hypoxia-induced injury in cardiomyocytes, AC16 cardiomyocytes were exposed to 48 h of culturing in hypoxic conditions.

### Cell Transfection

Transfection involved mixing 12 µl Lipofectamine 3000 (Invitrogen, Carlsbad, CA, USA) with 150 µl serum-free medium, adding 10 µl miR-449b-5p mimics (50 nM for overexpression) or inhibitor (100 nM for knockdown), and incubating at 25 °C. The solution was added to AC16 cardiomyocytes (70–80% confluence) in 6-well plates. To assess miR-449b-5p’s impact on BCL2L13, we co-transfected AC16 cardiomyocytes with 60 nM of miR-449b-5p mimics and 2 µg of pcDNA3.1-BCL2L13 or a control vector. These transfections were carried out for 48 h prior to subjecting the AC16 cardiomyocytes to hypoxic conditions.

### Cell Counting Kit-8 (CCK-8) Assay

Briefly, we seeded 3,000 AC16 cardiomyocytes per well in 96-well plates. After each treatment, we added 10 µl of CCK-8 reagent (cat. no. C0048S, Beyotime, Shanghai, China) to each well. Post a 2-h incubation at 37 °C, we measured the optical density (OD) at 450 nm using an Epoch microplate reader (BioTek, USA). Cell viability was calculated as: Cell viability (%) = (OD experimental group-OD blank) / (OD control-OD blank) × 100%.

### Lactate Dehydrogenase (LDH) Release Assay

Following various treatments, we lysed AC16 cardiomyocytes using 1% Triton X-100 solution (Sigma-Aldrich) for 20 min and then centrifuged at 400 x g for 5 min at 4 °C to discard cellular debris. We measured the LDH levels in the supernatant using a commercial LDH Kit (Beijing Solarbio Science & Technology Co., Ltd., cat. no. BC0685), following the provided instructions.

### Flow Cytometry

We determined apoptosis in AC16 cardiomyocytes using a FITC-Annexin V/PI kit (BD Biosciences, San Jose, CA, USA). In summary, following the collection and rinsing of cells with cold 1X PBS (pH = 7.4), they were incubated in the dark at room temperature for 15 min with a mixture containing binding buffer, FITC-Annexin V, and PI. We then analyzed the apoptotic cells using a FACS CantoII flow cytometer (BD Biosciences).

### Terminal Deoxynucleotidyl Transferase-mediated dUTP Nick-end Labeling (TUNEL) Staining

AC16 cardiomyocytes from different groups were directly cultured on cover slips. These were fixed in 1% formaldehyde for 10 min and permeabilized with 0.2% Triton X-100 for 2 min. The cells were then incubated with 50 µl of TUNEL reaction fluid (cat. no. C1086, Beyotime) for 1 h at 37 °C in a humidified chamber. Nuclei were stained using DAPI (cat. no. C1002, Beyotime). Photographs were captured using an FV1000 confocal laser scanning microscope. Apoptosis was quantified in five random fields per sample at 200× magnification, determined by the proportion of TUNEL-positive cells relative to the total number of nuclei​ [24].

### Evaluation of Oxidative Stress Indicators

We evaluated oxidative stress by measuring malondialdehyde (MDA, ab287797), reactive oxygen species (ROS, ab287839), and superoxide dismutase (SOD, ab178012) levels in the cell supernatants. This was done using specific commercial kits (Abcam, Cambridge, MA, USA), following the manufacturer’s protocols.

### Reverse Transcription-Quantitative PCR (RT-qPCR)

Total RNA was extracted from treated AC16 cardiomyocytes using TRIzol reagent (Invitrogen) as per the manufacturer’s instructions. RT-qPCR analyses were then performed on an ABI-7500 Fast Real-Time PCR system (Thermo Fisher Scientific, Inc.) using Taqman Universal Master Mix II (Thermo Fisher Scientific, Inc.) and Takara RR820B SYBR® Premix Ex Taq™ II. The amplification thermocycling conditions included an initial denaturation at 94°C for 30 s, followed by 30 cycles of annealing at 62°C for 10 s and extension at 70°C for 30 s. Primers for miR-449b-5p forward, 5’-GCCGAGAGGCAGTGTATTGTTA-3’ and reverse, 5’-AGGCAGTGTATTGTTAGCTGGC-3’; U6 forward, 5’-CTTCGGCAGCACATATAC-3’ and reverse, 5’-GAACGCTTCACGAATTTGC-3’; BCL2L13 forward, 5’-GCCAATTCAATGGCGTCCTC-3’ and reverse, 5’-TCAAAGCCTGTGCTGGTGAA-3’ and GAPDH forward, 5’-ACATGTTCCAATATGATTCC-3’ and reverse, 5’-TGGACTCCACGACGTACTCAG-3’ were utilized. The relative expression of miR-449b-5p and BCL2L13 mRNA was quantified using the 2-ΔΔCt method [25], normalized against U6 and GAPDH respectively.

### Bioinformatics Analysis and Luciferase Reporter Assay

Based on the predicted miR-449b-5p binding site on BCL2L13 identified via Targetscan7.1 (http://www.targetscan.org/vert_71/), we validated their interaction using a luciferase reporter assay. The wild-type (WT) and mutant (MUT) BCL2L13 binding sites for miR-449b-5p were then cloned into psiCHECK-2 vectors (Promega Corporation) to create WT or MUT BCL2L13 reporter plasmids. AC16 cardiomyocytes, seeded in 24-well plates, were co-transfected with these plasmids and miR-449b-5p mimics or NC using Lipofectamine 3000 (Invitrogen, USA). A total of 48 h following transfection, the activities of Firefly and Renilla luciferases were measured using a Dual-Luciferase reporter assay kit (Promega Corporation). The relative luciferase activity for each sample was calculated as the ratio of Renilla luciferase activity to Firefly luciferase activity.

### Western Blot Analysis

Protein samples were acquired by lysing cells with RIPA buffer (Thermo Fisher Scientific, Inc.) followed by centrifugation at 12,000 ×g for 15 min at 4 °C to obtain the supernatant. The protein levels were measured using a BCA assay kit (Beyotime). We then separated 30 µg of each protein sample using 12–15% SDS-PAGE gels and transferred them onto PVDF membranes. Following a blocking step using 5% non-fat milk, the membranes were incubated with primary antibodies targeting BCL2L13 (1:5000, ab203516), Bcl-2 (1:1000, ab196495), Bax (1:2000, ab182733), PI3K (1:2000, ab133595), p-PI3K (1:500, ab182651), AKT (1:500, ab8805), p-AKT (1:1000, ab8933), and GAPDH (1:2500, ab9485) from Abcam (Cambridge, UK). Subsequently, the membranes were incubated for 2 h at room temperature with horseradish peroxidase-linked secondary antibodies (1:5000, SC-2054, Santa Cruz Inc., Santa Cruz, CA, USA). Protein bands were detected using an enhanced chemiluminescence reagent (GE Healthcare, Braunschweig, Germany).

### Statistical Analysis

Data analysis was performed using GraphPad Prism 6.0 software (GraphPad Software, Inc.), and the results were reported as mean ± standard deviation (SD) from three separate experiments. For comparisons between two groups, Student’s t-test was utilized, whereas for comparisons among multiple groups, one-way ANOVA followed by either Dunnett’s or Tukey’s test was applied. A *p*-value below 0.05 was deemed to indicate statistical significance.

## Results

### Hypoxia-induced Cardiomyocyte Damage and Reduced miR-449b-5p Expression

**Fig. 1:**
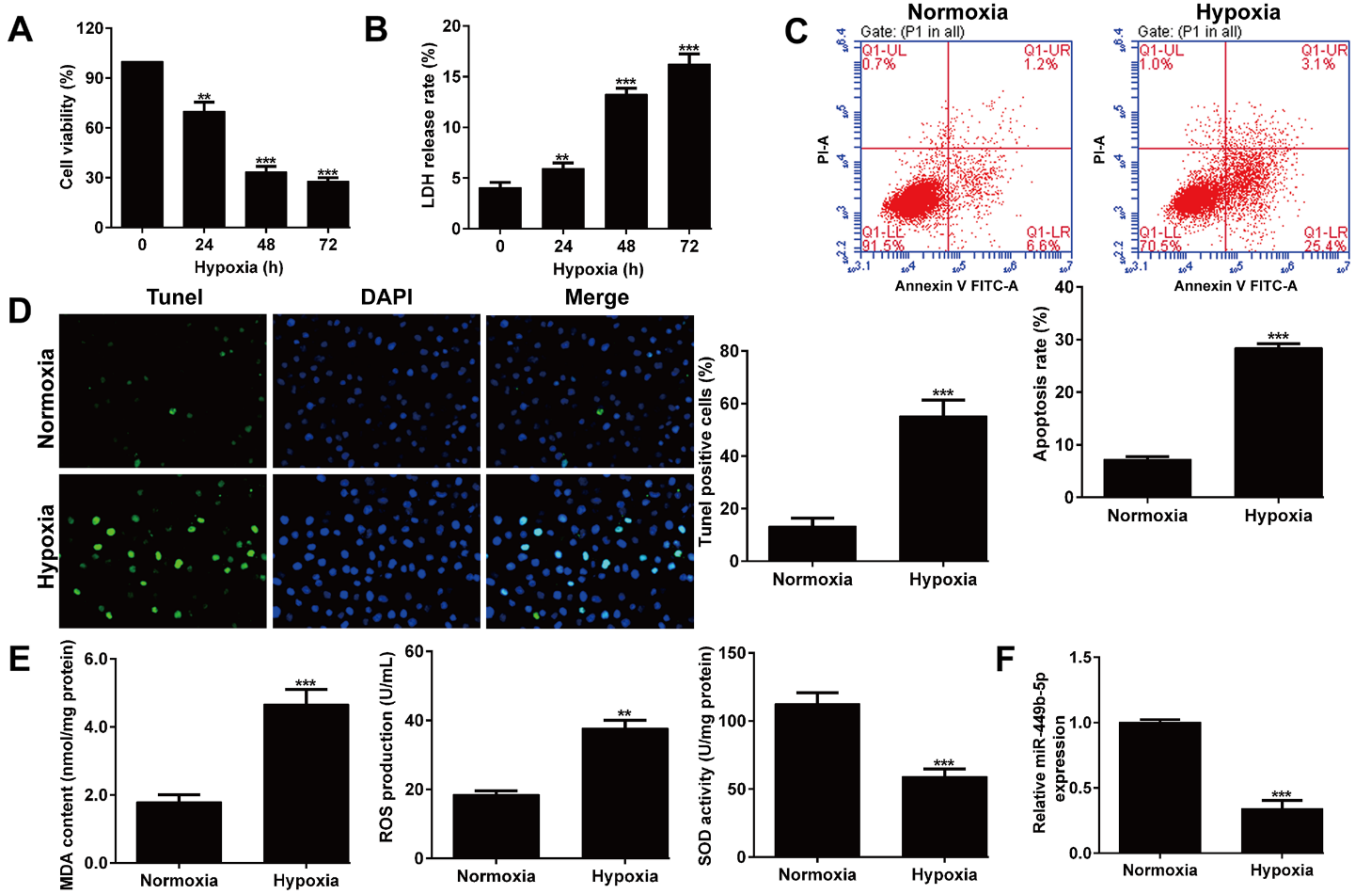
Expression levels of miR-449b-5p in hypoxic-induced AC16 cardiomyocytes. (**A**) CCK-8 assay was conducted to evaluate cell viability in AC16 cardiomyocytes after treatment with hypoxia for 0, 24, 48, and 72 h. Data analysis was conducted by one-way ANOVA, followed by Dunnett’s test. (**B**) The LDH release level was analyzed in AC16 cardiomyocytes after treatment with hypoxia for 0, 24, 48, and 72 h. Data analysis was conducted by one-way ANOVA, followed by Dunnett’s test. (**C**) Apoptosis of AC16 cardiomyocytes under 48 h hypoxia treatment was evaluated by flow cytometry with Annexin V/PI staining. (**D**) Representative images (left panel) of TUNEL staining of cardiomyocytes showing the apoptotic cells (200×; scale bar, 100 μm) and statistical results of TUNEL-positive cells per field (right panel; *n* = 3). (**E**) MDA content, ROS production and SOD activity were determined in AC16 cardiomyocytes under 48 h hypoxia treatment. (**F**) RT-qPCR was utilized to evaluate the expression level of miR-449b-5p in AC16 cardiomyocytes under hypoxia versus normoxia control. Data analysis was conducted by Student’s t-test. Bar graphs were shown as mean ± SD (*n* = 3). **p < 0.01, ***p < 0.001, compared with control

In our study, we first established an in vitro model of hypoxia-induced injury using human AC16 cardiomyocytes. Upon evaluating the cells under hypoxic conditions, we observed a time-dependent decrease in cell viability and a corresponding increase in LDH release, indicating increased injury (Fig. 1A and B). The 48-h hypoxic exposure was chosen as the optimal time point for significant viability reduction and LDH elevation. Flow cytometry and TUNEL staining revealed a significant increase in the apoptotic rate of AC16 cardiomyocytes under hypoxia compared to normoxic conditions (Fig. 1C and D). We also assessed oxidative stress and found elevated MDA and ROS levels, with a concurrent decrease in SOD activity in hypoxia-exposed cells (Fig. 1E). Importantly, RT-qPCR results showed a significant downregulation of miR-449b-5p in the hypoxia-treated cardiomyocytes (Fig. 1F). These findings suggest a pivotal role of miR-449b-5p in hypoxia-induced cardiomyocyte injury.

### MiR-449b-5p Mitigates Hypoxia-induced Damage in AC16 Cardiomyocytes

**Fig. 2:**
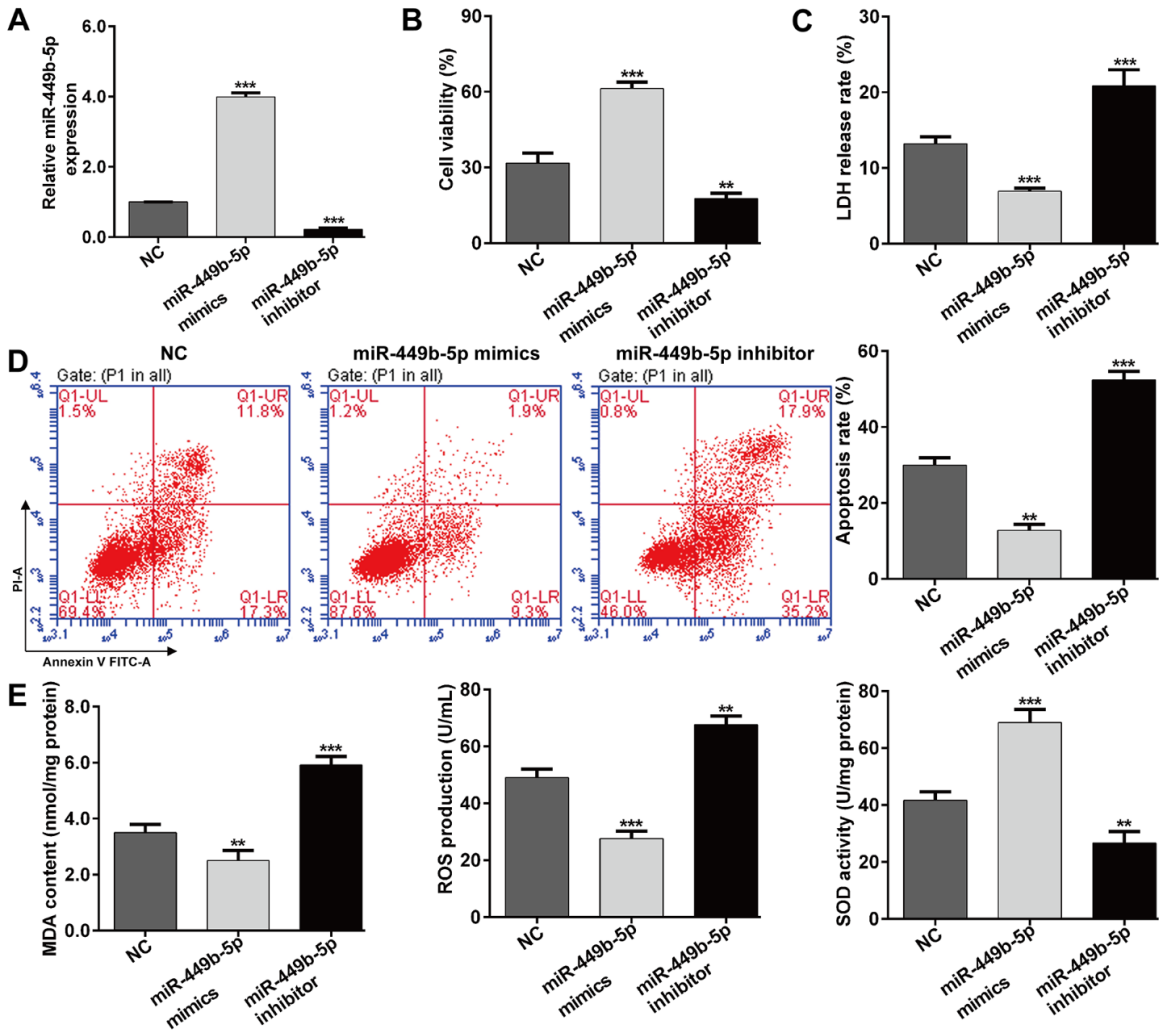
The regulatory role of miR-449b-5p on hypoxic-induced cardiomyocyte injury. AC16 cardiomyocytes were transfected with miR-449b-5p mimics or inhibitor for 48 h, followed by 48 h hypoxia treatment. (**A**) RT-qPCR determined miR-449b-5p levels in AC16 cardiomyocytes. (**B**) Cell viability and (**C**) LDH release levels were determined in AC16 cardiomyocytes. (**D**) Flow cytometry evaluated apoptotic cells in Annexin V-FITC+/PI + and Annexin V-FITC+/PI − quadrants in AC16 cardiomyocytes. (**E**) MDA content, ROS production and SOD activity were determined in AC16 cardiomyocytes. Data analysis was conducted by one-way ANOVA, followed by Tukey’s test. Bar graphs were shown as mean ± SD (*n* = 3). **p < 0.01, ***p < 0.001, compared with NC

The role of miR-449b-5p in the hypoxia-induced injury of AC16 cardiomyocytes was explored through both gain-of-function assays, employing miR-449b-5p mimics, and loss-of-function experiments, involving transfection with a miR-449b-5p inhibitor. Post-transfection efficiency was verified by RT-qPCR (Fig. 2A). Functional assays in hypoxic AC16 cardiomyocytes revealed that miR-449b-5p mimics reversed the hypoxia-induced reduction in cell viability, while the inhibitor exacerbated it (Fig. 2B). Overexpressing miR-449b-5p decreased LDH release, whereas its knockdown increased LDH release under hypoxic conditions (Fig. 2C). Flow cytometry showed that miR-449b-5p overexpression suppressed apoptosis, and its inhibition promoted apoptosis in these cells (Fig. 2D). Moreover, miR-449b-5p overexpression reduced MDA content and ROS production, and increased SOD activity, contrasting with the effects of miR-449b-5p knockdown under hypoxia (Fig. 2E).

### BCL2L13 Identified as a Target Gene of miR-449b-5p

**Fig. 3:**
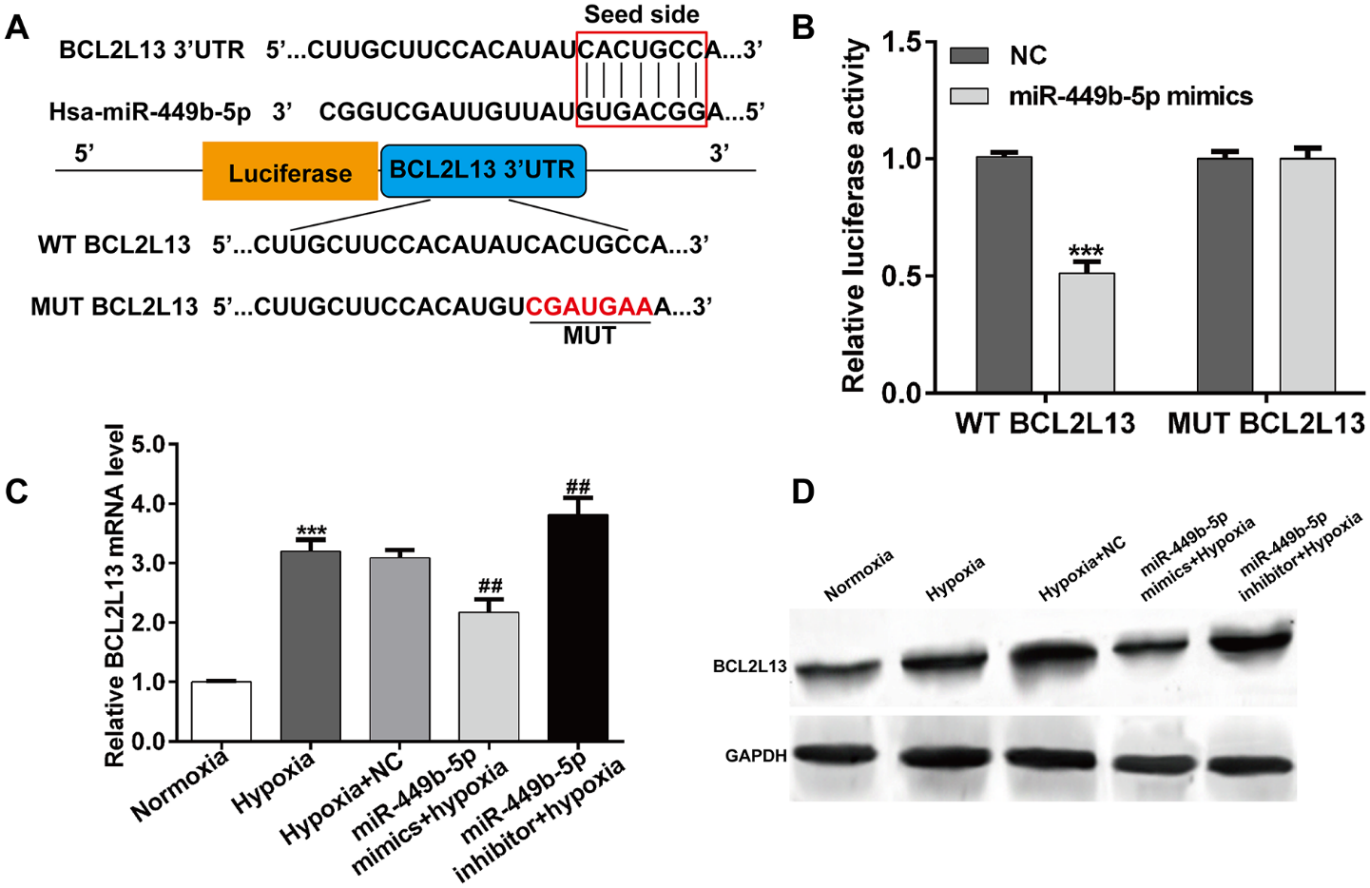
The relationship between miR-449b-5p and BCL2L13. (**A**) Predicted binding site of miR-449b-5p in the 3′ UTR region of BCL2L13. (**B**) The relative luciferase activity in AC16 cardiomyocytes by cloning a fragment of the wild-type (BCL2L13-WT) or mutant (BCL2L13-MUT) 3′-UTR of BCL2L13 into the luciferase reporter vector and followed by co-transfection with miR-449b-5p mimics or the negative control (NC). Data analysis was conducted by Student’s t-test. ***p < 0.001, compared with NC; (**C**) RT-qPCR detection of BCL2L13 mRNA expression and western blot analysis of BCL2L13 protein expression in AC16 cardiomyocytes. Data analysis was conducted by one-way ANOVA, followed by Tukey’s test. Bar graphs were shown as mean ± SD (*n* = 3). ***p < 0.001, compared with NC; ##*p* < 0.01, compared with hypoxia + NC

Targetscan 7.1 predicted a miR-449b-5p binding site in the 3′-UTR of BCL2L13 mRNA (Fig. 3A). To validate this interaction, we conducted a luciferase reporter assay. Results showed that co-transfecting miR-449b-5p mimics with WT BCL2L13 reporter plasmid significantly reduced luciferase activity, an effect not observed with the MUT BCL2L13 reporter (Fig. 3B). Additionally, RT-qPCR (Fig. 3C) and western blot analyses (Fig. 3D) revealed an increase in BCL2L13 expression under hypoxia, which was decreased by miR-449b-5p overexpression and increased with miR-449b-5p knockdown in AC16 cardiomyocytes. These findings suggest that miR-449b-5p downregulates BCL2L13 by directly binding to its 3’UTR.

### BCL2L13 Knockdown Mimicked miR-449b-5p’s Inhibitory Impact on Hypoxia-induced Cardiomyocyte Injury

**Fig. 4:**
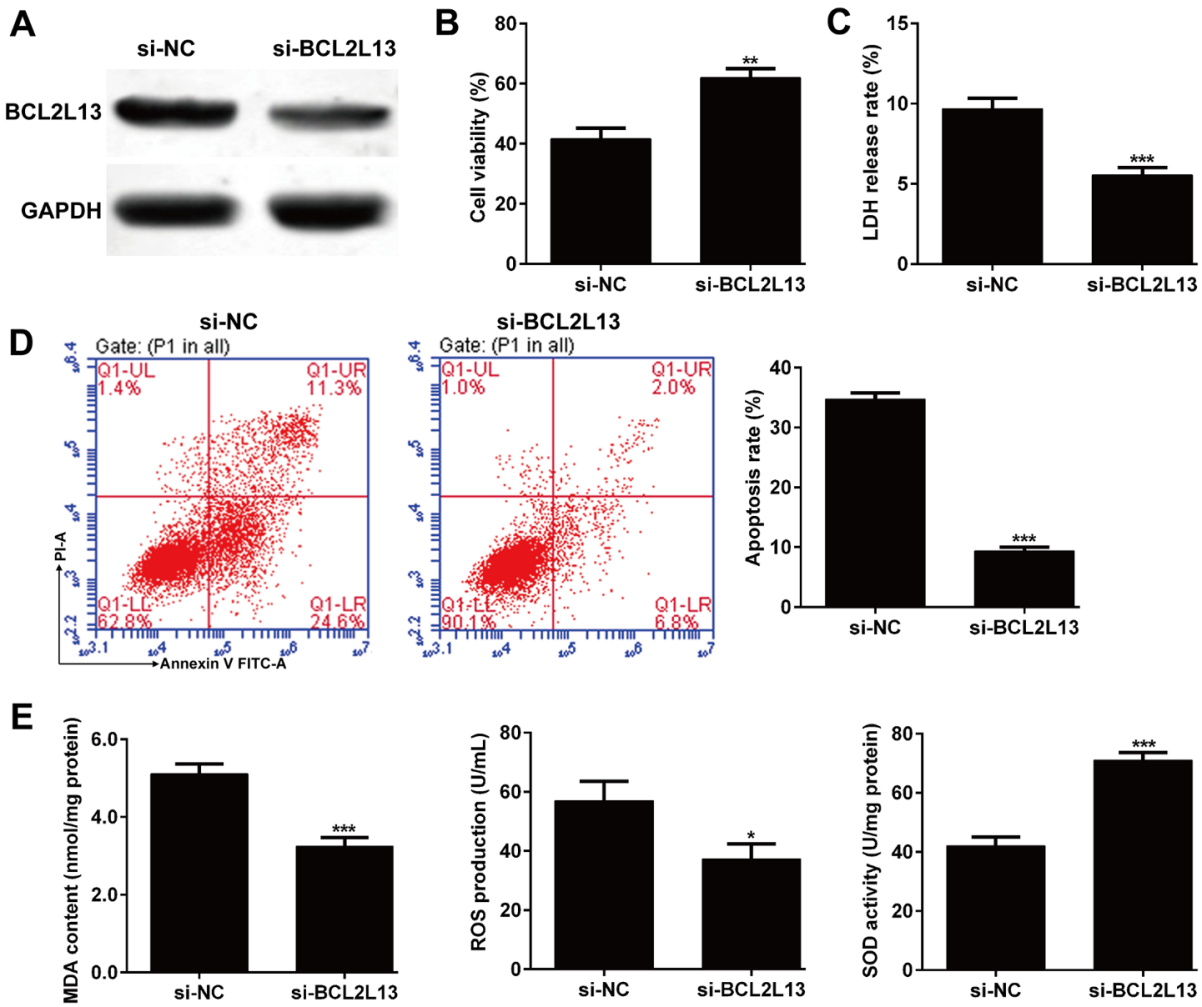
The regulatory role of BCL2L13 knockdown on hypoxic-induced cardiomyocyte injury. AC16 cardiomyocytes were transfected with si-BCL2L13 or si-NC, followed by 48 h hypoxia treatment. (**A**) Western blot analysis of BCL2L13 protein expression in AC16 cardiomyocytes. (**B**) Cell viability and (**C**) LDH release levels were determined in AC16 cardiomyocytes. (**D**) Flow cytometry evaluated apoptotic cells in Annexin V-FITC+/PI + and Annexin V-FITC+/PI − quadrants in AC16 cardiomyocytes. (**E**) MDA content, ROS production and SOD activity were determined in AC16 cardiomyocytes. Data analysis was conducted by Student’s t-test. Bar graphs were shown as mean ± SD (*n* = 3). *p < 0.05, **p < 0.01, ***p < 0.001, compared with si-NC

Given the observed upregulation of BCL2L13 in hypoxia-stressed AC16 cardiomyocytes, we conducted loss-of-function assays to explore BCL2L13’s role in hypoxia-induced cardiomyocyte injury. Western blot analysis confirmed the effective downregulation of BCL2L13 protein levels in hypoxia-treated cells post-transfection with si-BCL2L13, as opposed to si-NC transfection (Fig. 4A). Subsequent functional assays revealed that BCL2L13 knockdown notably enhanced cell viability (Fig. 4B), decreased LDH release (Fig. 4C), and reduced cell apoptosis (Fig. 4D) in hypoxic AC16 cardiomyocytes. Additionally, BCL2L13 suppression significantly lowered MDA content and ROS production, while increasing SOD activity in these cells (Fig. 4E).

### Reintroduction of BCL2L13 Nullified miR-449b-5p’s Protective Effects in Hypoxia-induced Cardiomyocyte Damage

**Fig. 5:**
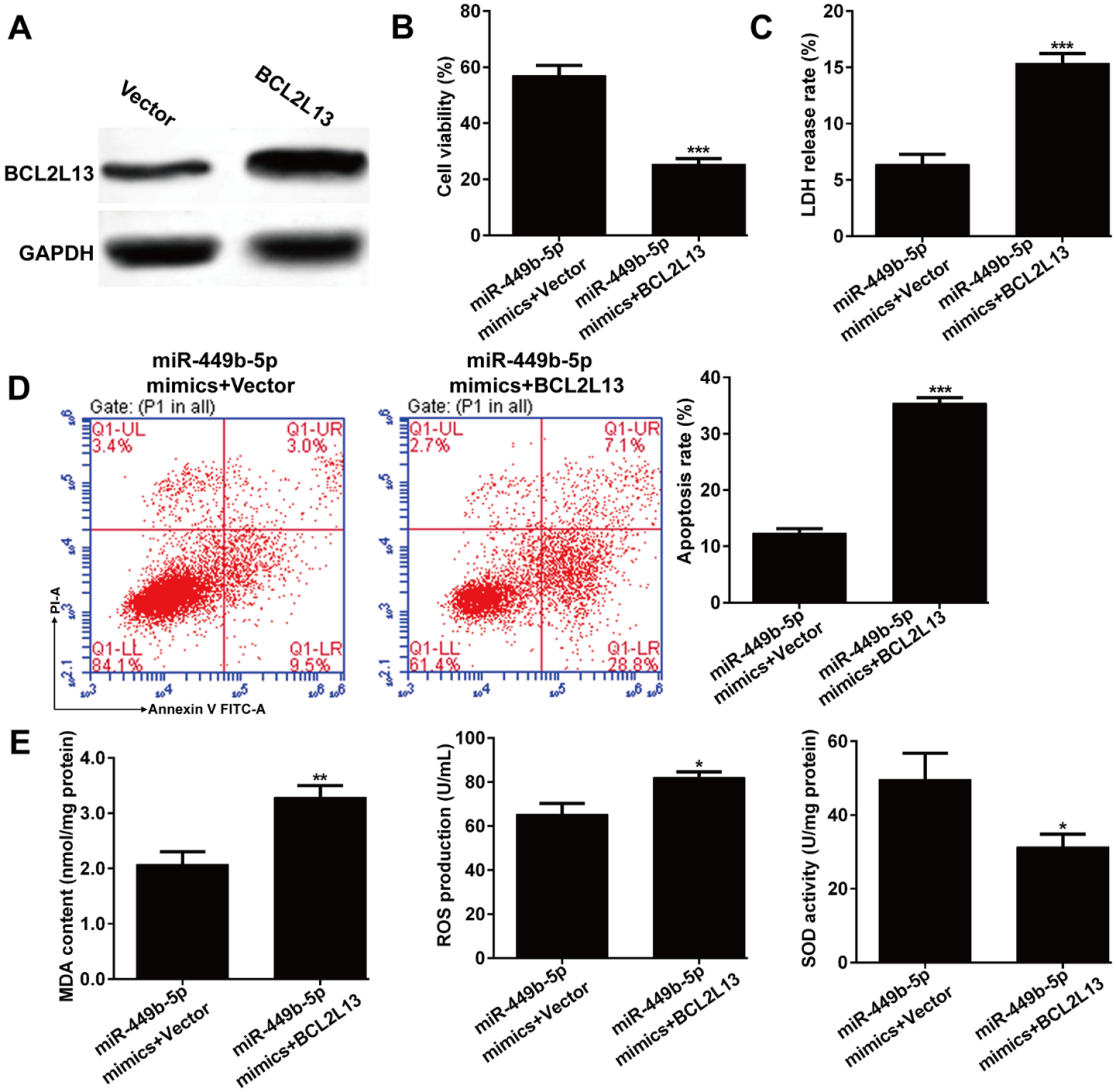
Validation of the role of BCL2L13 in the effects of miR-449b-5p overexpression on hypoxic-induced cardiomyocyte injury. AC16 cardiomyocytes were transfected with pcDNA3.1-BCL2L13 or empty vector, followed by 48 h hypoxia treatment. (**A**) Relative protein expression levels of BCL2L13 in AC16 cardiomyocytes by Western blot. (**B**) Cell viability and (**C**) LDH release levels were determined in AC16 cardiomyocytes. (**D**) Flow cytometry evaluated apoptotic cells in Annexin V-FITC+/PI + and Annexin V-FITC+/PI − quadrants in AC16 cardiomyocytes. (**E**) MDA content, ROS production and SOD activity were determined in AC16 cardiomyocytes. Data analysis was conducted by Student’s t-test. Bar graphs were shown as mean ± SD (*n* = 3). *p < 0.05, **p < 0.01, ***p < 0.001, compared with miR-449b-5 mimics + Vector

To understand if BCL2L13 modulates the miR-449b-5p-mediated effects on hypoxia-induced cardiomyocyte injury, we conducted rescue experiments. Overexpression of BCL2L13 in hypoxic AC16 cardiomyocytes, confirmed by Western blot (Fig. 5A), significantly reversed the miR-449b-5p mimic-induced improvements in cell viability and reductions in LDH release, as observed in CCK-8 (Fig. 5B) and LDH assays (Fig. 5C). Flow cytometry analysis indicated that the reduction in apoptosis caused by miR-449b-5p overexpression was notably negated by BCL2L13 overexpression (Fig. 5D). Moreover, BCL2L13 upregulation countered the effects of miR-449b-5p mimics on MDA content, ROS production, and SOD activity in these cells (Fig. 5E). This suggests that miR-449b-5p’s protective role in hypoxia-induced cardiomyocyte injury may involve targeting BCL2L13.

### MiR-449b-5p-mediated Activation of the PI3K/AKT Pathway through Targeting BCL213 in Cardiomyocytes

**Fig. 6:**
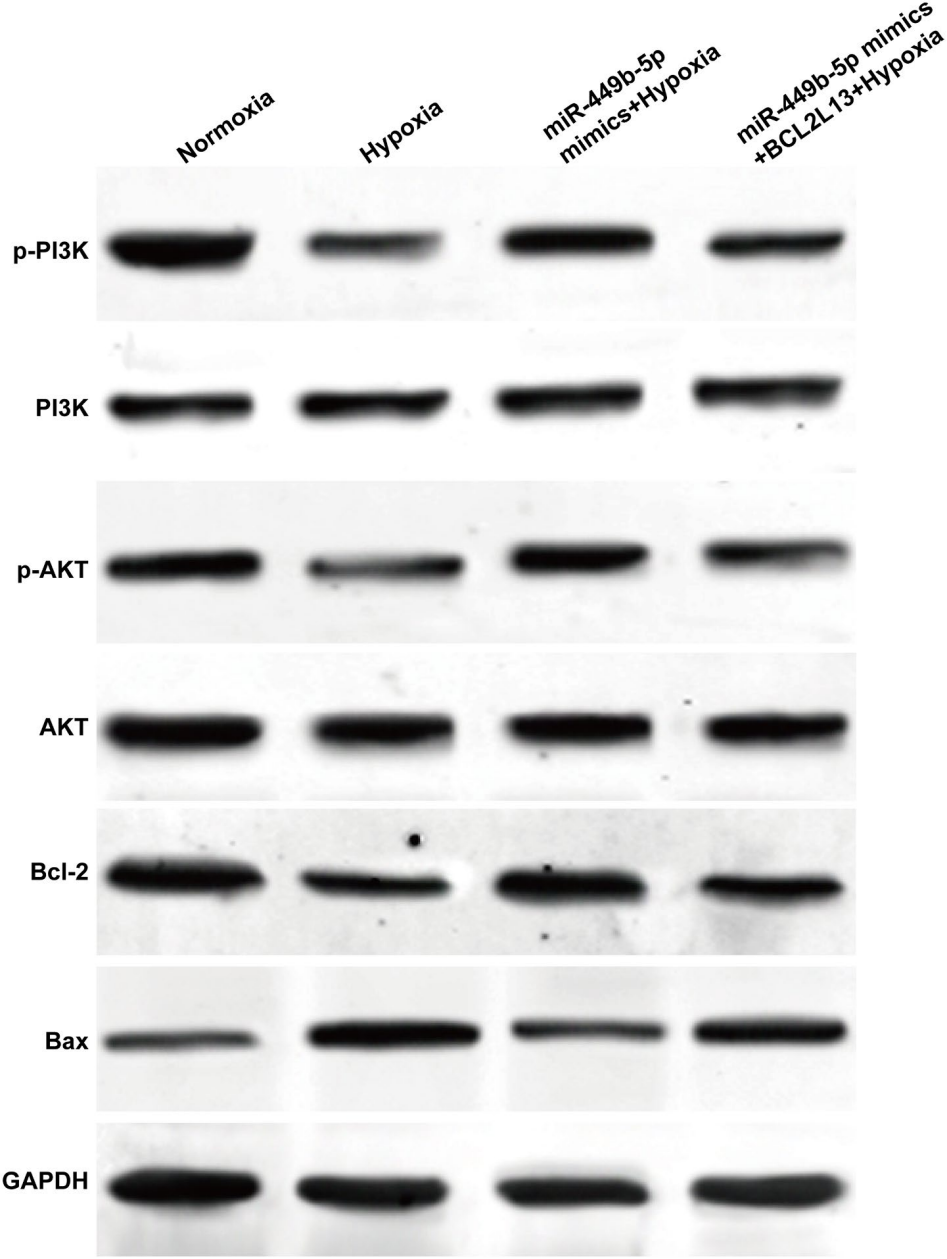
The regulatory role of miR-449b-5p/BCL2L13 axis on PI3K/AKT pathway. Western blot analysis was performed to measure the protein levels of p-PI3K, PI3K, p-AKT, AKT, Bcl-2 and Bax in hypoxic-induced AC16 cardiomyocytes after transfection with miR-449b-5p mimics alone or co-transfection with miR-449b-5p mimics + BCL2L13

Investigating the mechanism of miR-449b-5p in suppressing hypoxia-induced injury. We delved into the underlying mechanism through which miR-449b-5p mitigates hypoxia-induced injury by examining its impact on the PI3K/AKT pathway. Western blot analysis (Fig. 6) revealed that, under hypoxic conditions, there was a noticeable decrease in the expression levels of p-PI3K, p-AKT, and Bcl-2, coupled with an increase in Bax expression in AC16 cardiomyocytes. However, these alterations were significantly reversed upon miR-449b-5p overexpression. Intriguingly, co-transfection with BCL2L13 negated the effects of miR-449b-5p mimics on these protein expression levels, highlighting the pivotal role of the miR-449b-5p/BCL2L13 axis in modulating the PI3K/AKT pathway during hypoxia-induced injury.

## Discussion

In our research, we developed a cell-based model of myocardial ischemia by exposing AC16 cardiomyocytes to 48 h of hypoxic conditions. During this period, there was a notable decrease in the expression of miR-449b-5p within the AC16 cardiomyocytes. This decline in miR-449b-5p levels was associated with a reduction in cell survival, along with elevated rates of apoptosis and oxidative stress in the AC16 cardiomyocytes. Previous research has consistently highlighted that cardiomyocyte apoptosis and oxidative stress are primary contributors to myocardial injury [26, 27]. Hypoxia, a crucial pathological factor, has been shown to induce cardiomyocyte apoptosis and oxidative stress [28]. Our findings align with previous studies. For instance, Zhang et al. [14] observed a decrease in miR-449b-5p levels in patients following liver transplantation and in L02 cells subjected to hypoxia-reoxygenation (H/R). Additionally, Xu et al. [16] observed decreased miR-449b-5p expression in renal tissues and HK-2 cells subjected to I/R injury. Given these findings, we hypothesize that miR-449b-5p could act as a protective agent in preventing injury to cardiomyocytes caused by hypoxia.

To test our hypothesis, we conducted experiments to investigate the role of miR-449b-5p in hypoxia-induced AC16 cardiomyocytes. Our research revealed that increasing the levels of miR-449b-5p in these cells contributed to enhanced cell survival, decreased release of LDH, and reduced rates of cell apoptosis and oxidative stress. On the other hand, diminishing miR-449b-5p levels had adverse effects. These observations indicate that miR-449b-5p acts protectively, mitigating the damage inflicted by hypoxia on AC16 cardiomyocytes. Significantly, miR-449b-5p is also involved in controlling cell apoptosis across different diseases. For instance, in hypoxic glioma samples, miR-449b-5p was found to be downregulated, and its overexpression promoted cell apoptosis in glioma cells exposed to hypoxia [29]. Likewise, the overexpression of miR-449b-5p led to increased cell survival and diminished apoptosis in L02 cells exposed to H/R conditions [14]. Moreover, transfection with a miR-449b-5p mimic decreased inflammation and apoptosis in HK-2 cells that were induced by I/R injury [16]. Additionally, the administration of anti-miR-449b or APN has been reported to preserve cardiac Nrf-1 expression, mitigate cardiac oxidative stress, reduce apoptosis, minimize infarct size, and enhance cardiac function [18]. While the role of miR-449b-5p in oxidative stress has not been extensively studied, it’s reasonable to speculate that the reduced cell apoptosis observed with miR-449b-5p overexpression may be associated with decreased accumulation of ROS. ROS accumulation can disrupt mitochondrial function by reducing mitochondrial membrane potential, ultimately leading to mitochondrial dysfunction [30]. From these results, it can be inferred that miR-449b-5p has inhibitory effects on hypoxia-induced injury in cardiomyocytes by lessening apoptosis and oxidative stress.

Furthermore, our study identified BCL2L13 as a target gene of miR-449b-5p. Further data showed that inhibiting BCL2L13 greatly inhibited hypoxia-induced cell viability loss, LDH release, cell apoptosis, and excessive production of oxidative stress, whereas increasing BCL2L13 negated miR-449b-5p’s protective impact in hypoxic AC16 cardiomyocytes. BCL2L13 is well-known for its central role in regulating apoptosis, a critical process in response to I/R injury [31, 32]. At the molecular level, miR-449b-5p upregulated Bcl-2 and downregulated Bax expression in hypoxia-induced AC16 cardiomyocytes by directly targeting BCL2L13. BCL2L13, alternatively referred to as Bcl-rambo, belongs to the Bcl-2 protein family, encompassing both anti-apoptotic (Bcl-2) and pro-apoptotic (Bax) components, essential for controlling the intrinsic apoptotic pathway [33, 34]. In agreement with our observations, earlier studies have demonstrated that BCL2L13 can promote apoptosis and decrease the viability of hippocampal neural stem cells when modulated by miR-124 and miR-137 [35]. MiR-874-3p has been found to mitigate ischemic injury through the downregulation of BCL2L13, indicating its potential as a therapeutic target for ischemic stroke [21]. Additionally, BCL2L13 knockdown has been reported to alleviate injury in cells exposed to OGD/R in various cell types [22, 36]. Moreover, BCL2L13, acting as a mitochondrial receptor, has been associated with promoting adipogenesis by increasing oxidative phosphorylation, which correlates with enhanced mitochondrial fusion and biogenesis [37]. From this perspective, we propose that BCL2L13 induced apoptosis may occur through a mitochondrion-dependent pathway mediated by ROS [38], of which miR-449-5p as the upstream regulator of BCL2L13 involved in hypoxia-induced cardiomyocyte injury. Our study further demonstrated that miR-449b-5p activates the PI3K/AKT pathway by targeting BCL2L13 in hypoxia-induced AC16 cardiomyocytes. The PI3K-AKT pathway, an essential intracellular signaling route, plays a key role in the regulation of cell growth, metabolism, and programmed cell death [39]. Numerous studies have shown that alleviation of hypoxic injury or injury induced by H/R in cells is associated with the activation of the PI3K/Akt signaling pathway [40–42]. In line with our results, previous research has demonstrated that miR-181c protects against heart failure by inhibiting cardiomyocyte apoptosis through the PI3K/Akt pathway [43]. Huangfu et al. [44] d have also shown that miR-145-5p inactivates the PI3K/Akt pathway, promoting the apoptosis of H9c2 rat myocardial cells. Thus, we hypothesize that miR-449b-5p enhances cell viability, suppresses apoptosis, and reduces oxidative stress in AC16 cardiomyocytes during hypoxia-induced injury by activating the PI3K/AKT signaling pathway via targeting BCL2L13.

Our study has certain limitations that should be acknowledged. Firstly, we exclusively employed the human cardiomyocyte cell line AC16 for our experiments. Future investigations should consider utilizing a broader range of cardiomyocyte cell lines to reinforce the reliability of our findings. Furthermore, our study is constrained by the absence of in vivo experiments. To address this limitation, our future research endeavors will focus on establishing an AMI rat model, allowing for a more comprehensive exploration of the mechanisms involved in our study.

In summary, our study revealed a decrease in miR-449b-5p expression in AC16 cardiomyocytes following hypoxia exposure. Significantly, our study is the first to demonstrate that the overexpression of miR-449b-5p alleviates hypoxia-induced damage in AC16 cardiomyocytes by activating the PI3K/AKT pathway via the targeting of BCL2L13. These findings offer valuable insights into the molecular mechanisms involved in the pathogenesis of AMI. The discovery of this mechanism holds promise for improving clinical diagnosis and therapeutic strategies for ischemic heart disease.

## Data Availability

The data sets utilized or analyzed in this study can be obtained from the corresponding author upon reasonable request.
